# Successful outcome in acute gastric volvulus in a low resource hospital setting in Bangladesh: A case report

**DOI:** 10.1016/j.ijscr.2020.06.006

**Published:** 2020-06-15

**Authors:** Bappy Basak, M. Tasdik Hasan, Jessica Stauber, Amit Sen Gupta, Safiqul Islam, S.M. Quamrul Akther

**Affiliations:** a Shaheed Suhrawardy Medical College & Hospital, Dhaka, Bangladesh; b Department of Psychological Sciences, University of Liverpool, United Kingdom; c University of TexasMedical Branch at Galveston, USA

**Keywords:** Case report, Organoaxial volvulus, Young patient, Bangladesh

## Abstract

**Introduction:**

Gastric volvulus is a rare medical emergency that can be fatal if not recognized early. Its rarity makes it quite challenging to diagnose but delaying in diagnosis and treatment can lead to fatal complications. Meticulous assessment and broadened differential diagnosis are thus crucial. Organoaxial volvulus, in which the stomach rotates 180 degrees around its long axis, is the most common subtype of gastric volvulus, occurring predominantly in infants and older adults.

**Presentation of the case:**

Here, we present a case of acute organoaxial gastric volvulus in a 17-year-old male presenting to a low-resource hospital in Bangladesh with severe upper abdominal pain and postprandial vomiting. Initial assessment revealed severe epigastric tenderness and mild dehydration. Plain abdominal x-ray showed a hugely distended bowel with single air fluid level and thoracic herniation of the stomach. The patient was resuscitated then sent to the operating theatre for urgent laparotomy and sleeve gastrectomy with anterior gastropexy. Treatment was successful, and the patient survived.

**Discussion:**

Acute gastric volvulus, a life-threatening surgical emergency can be treated successfully if identified timely. Though the CT scan is diagnostic, Borchardt’s triad can be helpful as well.

**Conclusion:**

This is the second consecutive case managed by the same surgeon in the same hospital, highlighting that high index of clinical suspicion is an important tool to diagnose this condition early. Timely diagnosis and treatment are essential to avoid fatal complications, denoting the importance of documenting such cases.

## Highlights

• Life-threatening surgical emergency in a 17-year-old boy with common symptoms, abdominal pain and vomiting.
• Likely to be misdiagnosed due to the rarity of the disease.
• High index of clinical suspicion is crucial. Borchardt’s triad can be very helpful in acute cases.
• Quickly identified and Successfully treated.
• Timely treatment can prevent grave complications.

## 1 Introduction

Acute gastric volvulus is a rare surgical emergency with high rate of non-operative mortality (30–50%) [1]. It most commonly occurs in infants less than 1 year and adults over 50 years [2]. Prompt diagnosis followed by urgent surgical intervention is crucial to avoid life-threatening complications associated with this condition [3]. A purely clinical diagnosis is challenging, though classical presentation, typically known as Borchardt’s triad involves severe upper abdominal pain and distension, unproductive vomiting, and difficulty in nasogastric (NG) tube insertion is present in 70% cases [1] making a handy tool for diagnosing acute gastric volvulus. Though CT abdomen and barium meal are considered standard diagnostic tests for gastric volvulus [3,4], these are not readily available in limited resource institutions in countries like Bangladesh. This case report thus details an inspiring story of careful physical examination and clinical evaluation, leading to prompt diagnosis and successful management of a rare medical emergency. Furthermore, previous documentation of the first case of gastric volvulus guided the surgeon and team to work in a timely manner. The presentation of this case was prepared in accordance with the SCARE criteria [5].

## 2 Case report

A 17-year-old male presented to the emergency department of Shaheed Suhrawardy Medical College & Hospital, Dhaka, Bangladesh with a one-day history of severe epigastric pain and episodes of vomiting that progressed to dry retching. Pain was sudden, severe, colicky, and non-radiating. It started during a highway journey, just after taking heavy meal, and exacerbated with movement. Pain was unrelieved by analgesics. The patient vomited a large amount of undigested food particles. He reported a similar episode of pain after a heavy meal 2 months prior.

Initial examination revealed tachycardia, tachypnoea, mild dehydration, and a tense, distended, severely tender epigastrium but otherwise no concerning signs. Nasogastric tube insertion was attempted several times, but all without success. The patient was kept nil by mouth and given intravenous crystalloids. Laboratory blood results were unremarkable, including normal amylase and liver function tests. Though our provisional diagnosis was bowel perforation, the presence of Borchardt’s triad, pain following heavy meal, and past history of similar episode kept gastric volvulus in consideration. With this rare and rapidly fatal diagnosis in mind, simultaneous investigations and preparations for surgery were promptly performed.

**Fig. 1:**
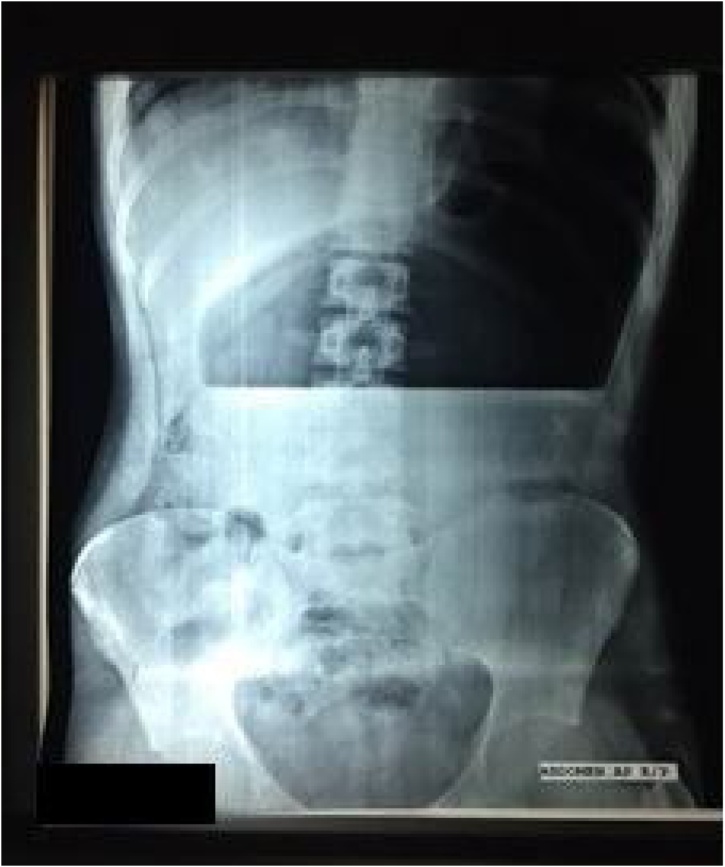
X ray abdomen reveals a hugely distended.

An abdominal X-ray, which was the most common investigation in our institution, showed a hugely distended bowel with large single air-fluid level in the abdomen (Fig. 1). It also showed herniation of gas containing gut in the thoracic cavity. After consulting with the radiology department, diagnosis of acute gastric volvulus was confirmed.

**Fig. 2:**
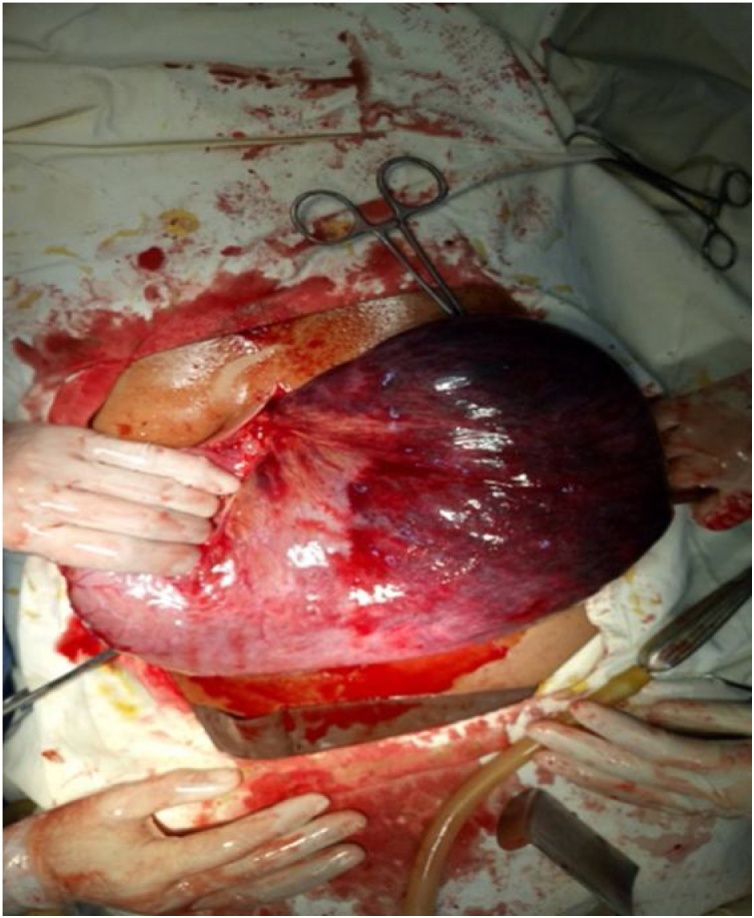
Necrosed fundus of acute gastric volvulus.

Our working diagnosis was acute gastric volvulus secondary to para-esophageal hiatal hernia. Patient was immediately transferred to the operation theatre for urgent laparotomy under general anesthesia. The abdomen was opened by midline incision. On per-operative findings, stomach was hugely distended and rotated along long axis. After derotation of the stomach fundus of the stomach was found necrosed predominantly along the greater curvature up to the gastro-esophageal junction (Fig. 2). Sleeve gastrectomy was done, and a feeding jejunostomy was kept in situ. After repairing the diaphragmatic hernia, anterior gastropexy was done.

**Fig. 3:**
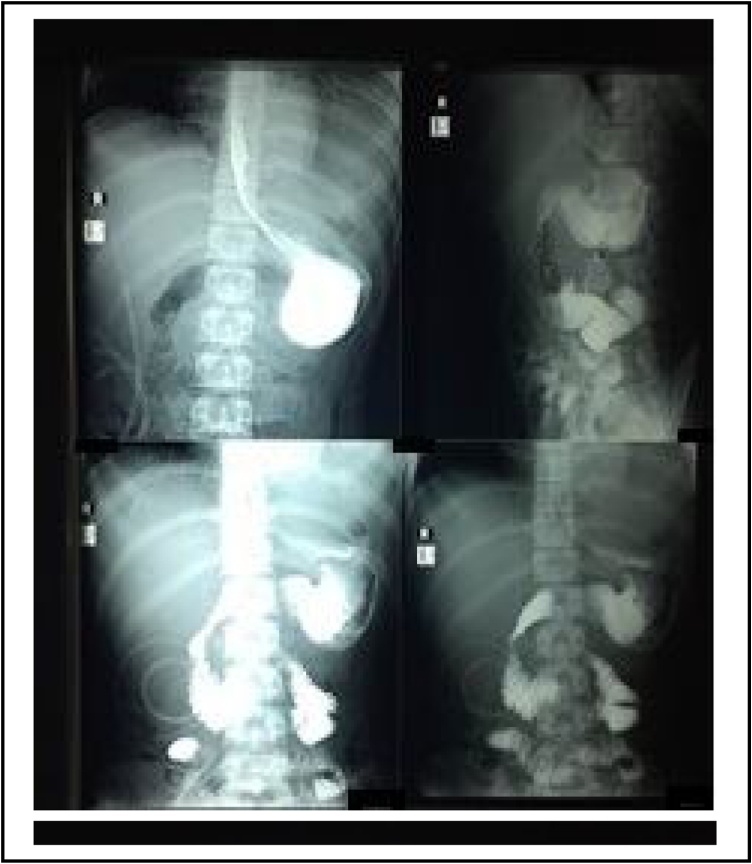
Contrast meal study of stomach and duodenum with fluoroscopy and spot diagnosis on 14th POD.

We performed a contrast meal study of stomach and duodenum with fluoroscopy and spot diagnosis on the 14th post-operative day (POD), which outlined partially resected stomach, duodenum, and part of the small intestine (Fig. 3). Mottle shadows were seen within opacified areas of the stomach. No ulcer, crater, or any mucosal abnormalities were identified. Initial gastric emptying was satisfactory. Duodenal cap was well-formed. The remaining duodenal loop appeared normal. Proximal jejunal loops were mildly dilated. Thick rogues were observed in the jejunum. After this report, slowly oral feeding was started in addition with jejunostomy tube feeding. Though the patient had some feeding difficulty in the early post-operative phase, he successfully advanced towards recovery throughout subsequent days.

Nasogastric tube was removed on 21th POD and feeding jejunostomy was kept in situ for 3 months. Patient eventually achieved full recovery from an otherwise acute, life-threatening emergency.

## 3 Discussion

Gastric volvulus is an acquired abnormal rotation of the stomach by more than 180 degrees, first described by Berti in 1866 and first surgically treated with success by Berg in 1897 [6]. It is very rare and usually presents after the fifth decade of life or in infants less than one year of age [2,7]. There is no sex or racial predilection [6].

Gastric volvulus can be classified based on axis rotation, severity (acute or chronic), extent (partial or total), and etiology (primary or secondary) [8]. The most frequently used classification is based on axis: Type 1 is organoaxial (rotation along the long axis) and accounts for 59% of cases, Type 2 is mesenteroaxial (rotation along the trans gastric or short axis) and accounts for 29% of cases, Type 3 is combined (combination of organoaxial and mesenteroaxial volvulus) and accounts for 2% cases, Type 4 is unclassified and accounts for 10% cases [8,9].

Primary causes are associated with tumors, adhesions, and ligament abnormalities whereas secondary causes are associated with disorders of gastric motility and anatomy or abnormalities with the diaphragm and spleen [10]. Secondary etiologies are more common, accounting for almost 70% of gastric volvulus cases [11]. In adults, para-esophageal hernia is the most common secondary cause, but can also result from trauma, eventration of the diaphragm, and phrenic nerve palsy [4,8].

The presentation of gastric volvulus can be acute, sub-acute or chronic. The classical Borchardt’s triad of retching, severe epigastric pain, and inability to pass NG tube is true for most acute cases, whereas subacute cases present with vague abdominal pain [10]. Chronic cases may present with non-specific findings such as dysphagia, dyspepsia, and intermittent postprandial pain, thus manifesting similarly to peptic ulcer disease, gastritis, cholecystitis, and atypical angina pectoris [4,12,13,14]. Use of Borchardt’s triad provides a valuable screening tool for gastric volvulus until proven otherwise, as exhibited by the presented case.

Gastric volvulus is considered a medical emergency and, if not promptly recognized, can lead to life-threatening complications including gastric ischemia, necrosis, and perforation [15]. Because of otherwise rich blood supply, stomach strangulation is uncommon in only occurs in 5%–28% of patients [6]. Patients suffering from a gastric infarction may present with gastrointestinal hemorrhage, cardiopulmonary failure, or shock [8]. Overall mortality is 30%–50% [16,17] for acute gastric volvulus, increasing to 60% if strangulation or infarction occurs.

The rarity of this disease makes early diagnosis difficult, thus requiring high index of clinical suspicion and confirmatory radiographic imaging [8]. In the case of gastric volvulus secondary to para-esophageal hernia, as reported here, the gastroesophageal junction remains in the abdomen, whereas the stomach ascends adjacent to the esophagus, resulting in an upside-down stomach that lies horizontally. X-ray findings include retrocardiac, gas/fluid-filled viscus on chest film, if the stomach is in the thorax, and a paucity of distal gas on plain abdominal film [1]. In chronic cases, especially those associated with para-esophageal hernia, barium meal is considered gold standard for diagnosis [7]. However, CT abdomen can also confirm the diagnosis and identify the transition point and should thus be the included in first-line investigation [18,19]. However, this can prove to be problematic in limited-resource settings like Bangladesh, as costs of diagnostic testing are always a concern when healthcare services are partially supported by the state.

Management of gastric volvulus varies according to presentation, cause, and patient-specific health factors. Acute gastric volvulus is a surgical emergency. Volume resuscitation, analgesics, and antiemetics should be initiated immediately, followed by insertion of a nasogastric tube to decompress the stomach (though it is quite difficult or impossible in these cases) [8]. It is important to note, however, that aggressive attempts to advance nasogastric tube may cause perforation especially in children [20].

Standard surgical approach involves emergency laparotomy with anterior gastropexy (fixation of the stomach to the anterior abdominal wall); however, partial or total gastrectomy may be required in cases of gastric necrosis or perforation [11]. In high-risk patients who are poor surgical candidates, endoscopic decompression and reduction can be considered [21]. Conservative management includes endoscopic reduction or percutaneous endoscopic gastrotomy tube insertion and is recommended in chronic cases, especially in the elderly [7]. Laparoscopic suture gastropexy is also safe and suitable for chronic gastric volvulus.

Other surgical approaches have also been mentioned in the literature, such as diaphragmatic hernia repair, gastropexy with diversion of gastrocolic ligament (Tanner’s operation), fundo-antral gastrostomy (Opolzer’s operation), and repair of diaphragmatic eventration [6]. Nissen’s fundoplication has also been done in cases of hiatal hernia [4].

In our previous case report of chronic gastric volvulus, anterior gastropexy with plication of the left diaphragm was done [22], whereas this acute case was treated with sleeve gastrectomy, feeding jejunostomy, anterior gastropexy with repair of the diaphragmatic hernia.

## 4 Conclusion

Because of the relative rarity of gastric volvulus [23,24], especially in adolescence and young adulthood, an acute episode is likely to be misdiagnosed or otherwise delayed in diagnosis. Recognizing symptoms with a high index of clinical suspicion is essential for early diagnosis, especially in a low-resource hospital setting where timely radiographic imaging is not easily accessible, if at all. Fatal consequences become inevitable if diagnosis is not made early enough to ensure timely intervention. Careful monitoring, quick diagnosis, and appropriate management can help prevent serious consequences. Documentation of this institution’s first case of gastric volvulus is what guided this team of surgeons to consider gastric volvulus as the prime differential diagnosis in the presented case. Hence, we hope that publishing cases such as this will help enable healthcare teams in other low-resource settings to clinically recognize gastric volvulus in a timely manner and, consequently, save multiple lives from fatality in such a rare, rapidly progressive, and otherwise treatable emergency.

## Declaration of Competing Interest

The authors declare that they have no conflict of interests.

## Funding

Not Such.

## Ethical approval

Ethical approval has been exempted by Institutional Review Board, Shaheed Suhrawardy Medical College and Hospital, Dhaka, Bangladesh considering the nature of the study.

## Consent

Written informed consent was obtained from the patient’s legal guardian for publication of the case report, any related images & diagnostic documents.

## Registration of research studies

1. Name of the registry: Not applicable.
2. Unique identifying number or registration ID: Not applicable.
3. Hyperlink to your specific registration (must be publicly accessible and will be checked):

## Guarantor

1) Dr S M Quamrul Akther, Associate Professor, Department of surgery, Shaheed Suhrawardy Medical College & Hospital, Sher-e- Bangla Nagar, Dhaka-1207, Dhaka, Bangladesh.
2) Dr Bappy Basak, Honorary Medical officer, Department of surgery, Shaheed Suhrawardy Medical College & Hospital, Sher-e- Bangla Nagar, Dhaka-1207, Dhaka, Bangladesh.

## CRediT authorship contribution statement

**Bappy Basak:** Conceptualization, Methodology, Data curation, Writing - original draft, Resources. **M. Tasdik Hasan:** Supervision, Resources, Validation. **Jessica Stauber:** Writing - review & editing. **Amit Sen Gupta:** Data curation. **Safiqul Islam:** Data curation. **S.M. Quamrul Akther:** Investigation, Supervision, Project administration.
